# Altered dynamic functional topology in first-episode untreated patients with schizophrenia can aid in early diagnosis

**DOI:** 10.1192/j.eurpsy.2022.321

**Published:** 2022-09-01

**Authors:** W. You, L. Luo, Q. Li, Y. Wang, Y. Wang, Q. Gong, F. Li

**Affiliations:** West China hospital of Sichuan university, Radiology, Chengdu, China

**Keywords:** Positive and Negative Syndrome Scale scores, schizophrénia, classification, dynamic functional topology

## Abstract

**Introduction:**

There is a growing consensus on brain networks that it is not immutable but rather a dynamic complex system for adapting environment. The neuroimaging research studying how brain regions work collaboratively with dynamic methods had demonstrated its effectiveness in revealing the neural mechanisms of schizophrenia.

**Objectives:**

To investigate altered dynamic brain functional topology in first-episode untreated schizophrenia patients (SZs) and establish classification models to find objective brain imaging biomarkers.

**Methods:**

Resting-state-functional magnetic resonance data for SZs and matched healthy controls were obtained(Table1).

**Figure:**
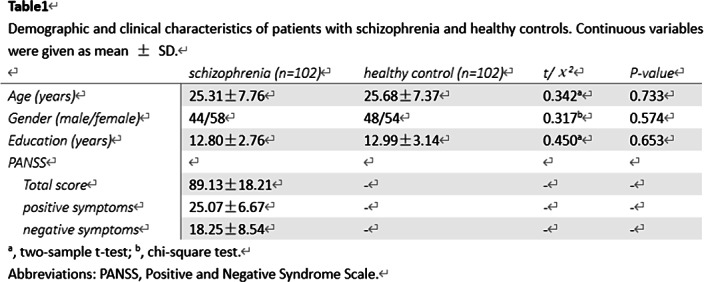

Power-264-template was used to extract nodes and sliding-window approach was carried out to establish functional connectivity matrices. Functional topology was assessed by eigenvector centrality(EC) and node efficiency and its time-fluctuating was evaluated with coefficient of variation(CV). Group differences of dynamic topology and correlation analysis between Positive and Negative Syndrome Scale(PANSS) scores and topology indices showing group differences, which also were used in establishing classification models, was examed.

**Results:**

The CV of node efficiency in angular and paracingulate gyrus was larger in SZs. There are 13 nodes assigned into several brain networks displaying altered CV of EC between groups(Figure1.A). Fluctuation of EC of the node in DMN, which was lower in SZs, showed negative correlation with PANSS total scores(Figure1.B). Dynamic functional topology of above nodes was used to train classification models and demonstrated 80% and 71% accuracy for support vector classification(SVC) and random forest(RF), respectively(Figure2).

**Figure:**
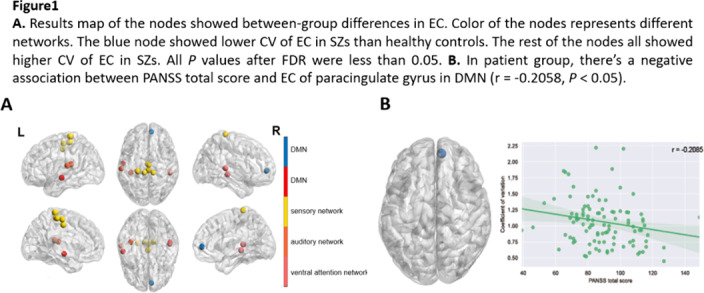

**Figure:**
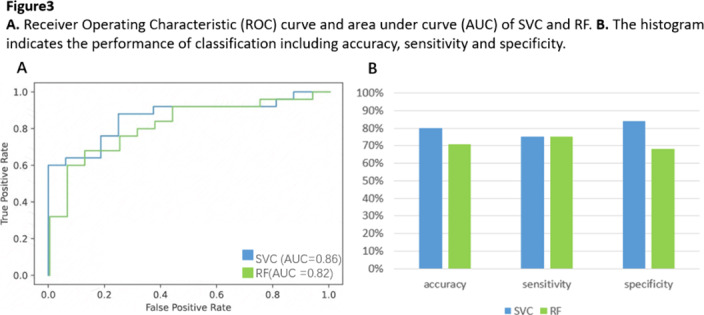

**Conclusions:**

Dynamic functional topology illustrated a capability in identifying SZs. Aberrated dynamics of DMN relevant to severity of patient’s symptoms could reveal the reason why it contributed to classification.

**Disclosure:**

No significant relationships.

